# A Systematic Literature Review on Depopulation Methods for Swine

**DOI:** 10.3390/ani10112161

**Published:** 2020-11-20

**Authors:** Andréia G. Arruda, Tariku J. Beyene, Justin Kieffer, Joshua N. Lorbach, Steven Moeller, Andrew S. Bowman

**Affiliations:** 1 Department of Veterinary Preventive Medicine, College of Veterinary Medicine, The Ohio State University, Columbus, OH 43215, USA; lorbach.5@osu.edu (J.N.L.); bowman.214@osu.edu (A.S.B.); 2 Center for Surgical Outcomes Research, The Research Institute at Nationwide Children’s Hospital, Columbus, OH 43215, USA; jibattariku@gmail.com; 3 Department of Animal Sciences, College of Food, Agricultural, and Environmental Sciences, The Ohio State University, Columbus, OH 43215, USA; kieffer.22@osu.edu (J.K.); moeller.29@osu.edu (S.M.)

**Keywords:** swine depopulation, systematic review, emergency preparedness

## Abstract

**Simple Summary:**

Mass depopulation involves ending the life of large numbers of animals due to emergency situations, for example, disease epidemics and environmental disasters. Depopulation should be conducted in a way that assures rapid and reliable unconsciousness followed by death. Our review article aimed to summarize depopulation methods available for swine and to highlight gaps in knowledge to guide the focus of future research. Our findings showed that the majority of research has been conducted with inhalable gaseous formulations (e.g., CO_2_) and that there was a lack of research in methods such as the use of captive bolts, electrocution, and oral formulations. Furthermore, our literature review showed a lack of research in the areas of worker safety, worker emotional health, and on validation of animal-based measures to be used for depopulation welfare assessments. Finally, a safe and reliable manner to induce unconsciousness and death for large populations of swine is lacking and urgently needed for preparedness purposes.

**Abstract:**

Swine mass depopulation refers to the destruction of large numbers of pigs and may include not only animals affected with a disease but also healthy pigs in a facility or surrounding areas. Emerging applications of mass depopulation include reducing welfare issues associated with slaughter delays, which was observed in the United States in 2020 as a result of the Coronavirus disease (COVID-19) pandemic. The objectives of this review were to summarize the available literature on swine depopulation methods and to highlight critical gaps in knowledge. Peer-reviewed articles were identified through a systematic search in electronic databases including Web of Science, MEDLINE, and PubMed. A total of 68 publications were assessed. Gaseous carbon dioxide inhalation was the most commonly reported depopulation method for both small- and large-scale trials. Measurements of consciousness state, which serves to assess suffering and humaneness, appeared to be lacking in a high proportion of the studies. None of the published studies demonstrated an ideally reliable and safe way to induce rapid unconsciousness in large groups of pigs. Development of rapid mass depopulation methods applicable to large groups of pigs is necessary to provide industry partners with suitable and low-cost emergency preparedness procedures while adhering to personnel safety and animal welfare standards. Lastly, there is an urgent need to standardize comprehensive reporting guidelines for depopulation studies.

## 1. Introduction

In the swine industry, mass depopulation refers to the rapid destruction of swine from one or more production sites in response to urgent circumstances. Historically, the most common reasons for depopulation have been immediate disease control, where termination of swine on the farm is necessary to minimize the risk of highly virulent pathogen spread; response to natural or human-made disasters; and, in the case of public health involvement, to protect from zoonosis [1,2,3,4,5]. Mass depopulation involves ending the life of large numbers of pigs and may not only include animals infected with a disease but also healthy pigs in facilities or surrounding areas. It is important to note that, even though euthanasia and humane slaughter methods can be used for the purposes of depopulation, not all depopulation methods are considered to be euthanasia by the American Veterinary Medical Association (AVMA) when the focus is ending the life of an individual animal in a way that minimizes pain and distress [6]. An example application of mass depopulation is ending the life of large numbers of swine due to the unanticipated disruption in access to packing plants observed in the US in 2020, which leads to welfare issues associated with inadequate space availability within barns for market-ready pigs. Coupled with closures of restaurants, hotels, and schools where pork products are consumed in large quantities, packing and processing plant closures, and delays due to the uncontained spread of the novel severe acute respiratory syndrome coronavirus 2 (SARS-CoV-2) during the COVID-19 pandemic have caused marketing disruptions throughout the food supply chain [7] that have required emergency depopulation.

The American Veterinary Medical Association (AVMA) classifies various pharmacological, physical, and electrical techniques, including injectable and gaseous agents, mechanical trauma, and electric shock as either ‘preferred’ and ‘preferred under constrained circumstances’ for depopulation of animals [1]. According to the AVMA, methods considered ‘preferred’ “are given the highest priority and should be utilized preferentially when emergency response plans are developed and when circumstances allow reasonable implementation during emergencies” [1]. In contrast, methods considered ‘permitted in constrained circumstances’ refer to methods permitted “only when the circumstances of the emergency are deemed to constrain the ability to reasonably implement a preferred method” [1]. In any case, once applied, the methods used should be able to result in the death of a complete population of animals in a quick and effective manner [1,8]. Ideally, this would occur rapidly and reliably by inducing unconsciousness followed by respiratory or cardiac arrest and, lastly, loss of brain function confirming the death. The absence of reflexes has been used as a standard metric to determine insensibility and to confirm brain death [9,10,11,12,13,14]. To induce insensibility, gaseous, physical, and injectable formulations should ultimately target one of the brain regions involved in consciousness, which include the cerebral cortex, the thalamus, and the brainstem [12]. In addition, techniques should minimize any stress and anxiety experienced by the animal prior to unconsciousness [15,16], reduce the need for individual animal handling and physical restraint, facilitate carcass containment and disposal transport, reduce contact of people with animals as well as the applied method, and be applicable to large groups of animals. 

The selection of a depopulation method varies based on the specific purpose of the method, age and number of pigs, available personnel, and availability of equipment and resources. The use of inhalable gaseous formulations, mainly carbon dioxide (CO_2_), appears to be the most studied depopulation method in the swine industry [3,13,17,18,19,20,21,22,23,24,25,26,27]. This method allows for multiple pigs to be euthanized at once, reduces the need for individual pig handling and instrument application, and is effective across all pig age categories. Despite these benefits, the use of CO_2_ for depopulation also has drawbacks including animal aversion and distress, and the need for appropriate equipment (e.g., vaporizers and push tanks for appropriate CO_2_ supply) [16,28].

Pilot and small-scale experimental studies have been conducted in the past to identify suitable swine depopulation methods in regard to equipment design, cost, and efficiency of attaining desirable outcomes including speed of unconsciousness or insensibility without raising animal welfare issues [25]. However, there is a need to compile outcomes and characteristics of various methods for evidence-based decision-making during specific field circumstances. Given the potential application of these methods for large-scale depopulation, the scientific community needs to explore improvements in mass depopulation methods applied under field conditions.

The objective of this review is to summarize available literature information and highlight critical gaps in knowledge pertaining to swine depopulation methods classified by the AVMA as ‘preferred’ and ‘permitted under constrained circumstances’.

## 2. Materials and Methods 

### 2.1. Article Search Strategy

Peer-reviewed articles were identified by systematic search in electronic literature databases including Web of Science/CAB Abstract/MEDLINE and PubMed on 11 May and 12 May 2020. The databases search included the following search title/abstract/keywords: (swine OR porcine* OR sow* OR pig* OR hog* OR boar) AND (depopulation OR stunning OR euthan*) addressing themes of swine euthanasia, mass depopulation, and stunning methods. Articles identified by the searches were uploaded into EndNote X9 reference manager and duplicates were automatically detected and removed [29] followed by manual removal of any additional duplicates (i.e., publications published in more than one format, or indexed in more than one database). A general protocol for this review was created a priori and critically assessed using the Assessment of Multiple Systematic Reviews (AMSTAR) measurement tool for the methodological quality of candidate peer review articles [30]. 

### 2.2. Article Screening and Selection Criteria

**Figure 1:**
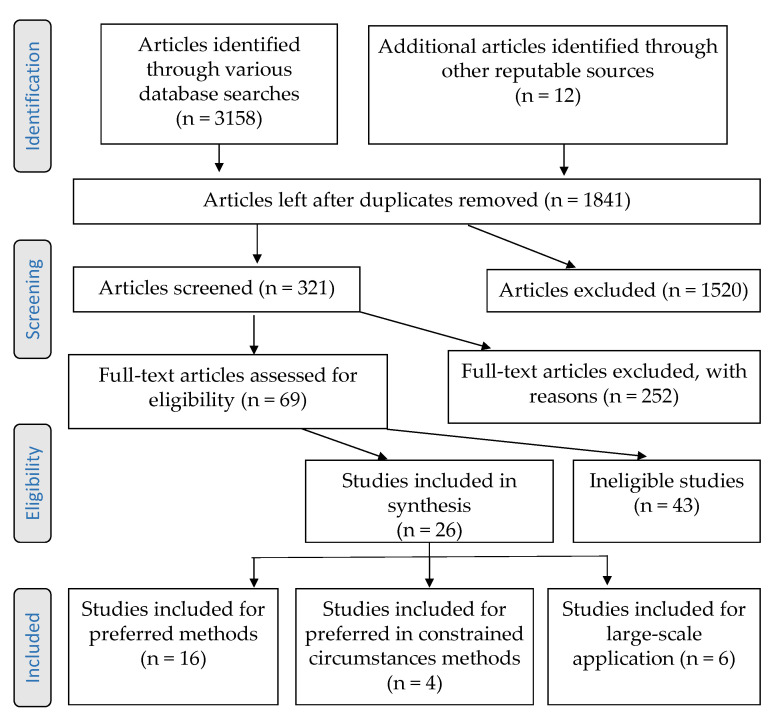
Summary of the literature searching and screening process using the Preferred Reporting Items for Systematic reviews and Meta-Analysis (PRISMA) checklist flow diagram.

Literature was screened systematically according to the schematic framework following the Preferred Reporting Items for Systematic reviews and Meta-Analysis (PRISMA) guidelines as shown in Figure 1 [31]. Peer-reviewed articles on euthanasia and or depopulation methods solely focusing on animal species other than swine, and publications in languages other than English, were excluded. Evaluations of each title and abstract were performed using inclusion criteria for article screening that included:

- Type of study: descriptive and analytical (observational and experimental) studies
- Date of publication: articles published between January 1990 and May 2020
- Geographic focus: worldwide
- Population: applied to individual pigs or groups
- Depopulation methods: “preferred” and “permitted under constrained circumstances” methods specifically for swine species as listed by AVMA Guidelines for the Depopulation of Animals [1]. These methods are summarized in Table 1, along with any other depopulation method identified in the search
- Outcomes/efficiency measurements: speed of unconsciousness, insensibility, and death

### 2.3. Article Assessment Process

For each selected article, a record was made in a Microsoft Excel spreadsheet (available from authors upon request) describing: (1) the geographical location where the study was conducted, (2) objective(s) of the study, (3) summary of the study, (4) method and chemical/physical agent used for depopulation and/or euthanasia, (5) number and age of pigs included in the study, (6) method of measurement for unconsciousness and speed of unconsciousness, (7) insensibility and death confirmation methods, (8) carcass disposal method, (9) human safety assessment, (10) personnel skill, equipment, and facilities requirements, and (11) limitations of method applied in the specific context. For quality assurance purposes, three studies were independently extracted by two authors to pilot-test the data extraction and the process was refined accordingly to ensure consistency between reviewers. Any disagreements were resolved by consensus.

## 3. Results

### 3.1. Overview of the Included Studies

The combination of search terms in the selected databases resulted in 3168 peer-reviewed articles and an additional 12 publications identified from grey literature from reputable sources including the AVMA, the OIE-World Organization for Animal Health, and the National Pork Board (NPB) (Figure 1). Ultimately, this review included 26 studies assessing AVMA preferred swine depopulation methods (*n* = 16), permitted in constrained circumstances swine depopulation methods (*n* = 4), or large-scale application of swine depopulation methods (*n* = 6). Out of the six large-scale studies, five used CO_2_ and one study used argon. 

### 3.2. Small-Scale Studies Using AVMA Preferred Depopulation Methods

Twelve experimental studies assessed different concentrations of gaseous carbon dioxide (CO_2_), nitrogen (N_2_), argon, and combinations in reducing respiratory distress in young and adult pigs during euthanasia or depopulation [13,17,18,19,20,21,22,23,28,32,33]. Three studies assessed the effectiveness of a non-penetrating captive bolt for euthanasia of newborn piglets [34,35,36]. One study was identified that includes each of the three remaining physical methods (i.e., gunshot, electrocution, and manual blunt force trauma) [23,35,37].

#### 3.2.1. Injectable Anesthetic Agents

Injectable anesthetic-type agents are preferred methods for individual animal euthanasia because of their rapid action and ability to induce a smooth transition to unconsciousness and death, which is a desirable outcome for the operator and observers [6]. Although there were no small or large-scale studies identified through the systematic literature search, Shearer and colleagues [38] listed drawbacks of using barbiturates and barbituric acid derivative for individual euthanasia to include: the high cost of the drug, the need for restraint during drug delivery, regulatory requirements specifying these agents need to be administered only by a licensed veterinarian, the need for a license for controlled substances for the veterinarian involved, and environmental safety requirements limiting carcass disposal options due to drug residues. In situations requiring euthanasia of a sizeable group of animals, it may be difficult to secure a sufficient volume of the drug to meet euthanasia needs and the method requires a great amount of time. For these reasons, injectable agents are not an ideal choice for executing swine mass depopulation, even though there are reports of its use for depopulation of approximately 2,460,000 young animals (3 to 17 day old) by lethal injection during the 1997–1998 Classical swine fever outbreak in The Netherlands [39,40].

#### 3.2.2. Inhaled Agents/Gas 

Four studies [13,17,23,28] assessed the efficacy of various concentrations of CO_2_ for euthanasia of both individual animals and groups of up to 658 pigs. The remaining eight studies [18,19,20,21,22,32,33] examined the efficacy of N_2_ only or CO_2_ for euthanasia in comparison to other methods, including N_2,_ CO_2_ mixture with argon, CO_2_ mixture with nitrogen, and prior exposure to nitrous oxide (N_2_O) followed by CO_2_ and electrocution. Approximately half of the studies were on young pre-weaned piglets while the rest were on adult pigs. 

Zhang [33] investigated the use of a cylindrical steel chamber for euthanasia of groups of 1, 3, 10, and 29 piglets (30 kg or smaller) using compressed N_2_ cylinders, with N_2_ introduced at varying rates. They captured both welfare and “public perception” related measurements and reported that rapid N_2_ introduction (reduction of O_2_ to <2% within 3 min) was effective in inducing unconsciousness quickly (within less than one minute) with minimal irritating behavior. However, they also reported longer-lasting seizures (up to four minutes) that may not be well received by the public. Their findings were similar across animal group sizes assessed.

Raj and Gregory [18] assessed two replicate groups of adult male pigs (55 to 72 kg) in separate trials containing six and ten animals each for their behavioral reluctance as they entered three gaseous atmospheres (90% argon, 30% CO_2_, or 90% CO_2_) to get a food reward. In both replicate groups, the total time to first withdrawal and time spent in the box containing 90% CO_2_ were significantly shorter compared to 30% CO_2_ and 90% argon, even after 16–24 h of fasting. The total time spent in the box during the 30% CO_2_ treatment was similar to the time recorded for the control days (regular air). However, in this descriptive study where pigs in the two groups were exposed to each gaseous treatment over a period of 10 days without changing groups across treatments over time, inhalation of 90% CO_2_ was aversive to the majority (88%) of pigs. Relatively minimal aversion was observed during inhalation of lower concentrations of CO_2_ (30%) and inhalation of 90% argon. 

Raj and Gregory [19] also evaluated the severity of respiratory distress just before loss of posture as a result of exposure to different concentrations of CO_2_ in combination with argon and O_2_. They evaluated 95 pigs (15 to 31 kg) and found that exposure to 40–70% CO_2_ in air induced severe respiratory distress, resulting in a considerable proportion (48.3%) of the pigs attempting to escape from the gas; whereas exposure to 80–90% CO_2_ resulted in no attempts to escape. Exposure to 90% CO_2_, in comparison with all the other treatments, resulted in a faster onset of loss of posture, while exposure to 30% CO_2_ in argon with 5% residual O_2_ resulted in the slowest time to loss of posture. They reported minimal respiratory distress in pigs exposed to 2% O_2_ in argon.

Similarly, Velarde et al. [17] and Verhoeven et al. [13] evaluated pigs individually for aversive behavior. Velarde et al. [17] compared aversion to the dip-lift stunning system and to inhalation of 70% and 90% CO_2_ in halothane gene-free and heterozygous halothane genotype slaughter weight pigs. Based on the number of attempted retreats, for the first descent into the well with atmospheric air, heterozygous halothane pigs were more averse to CO_2_ compared to halothane gene-free pigs. On repeating the descent, heterozygous halothane pigs showed greater habituation to the procedure. Behavioral aversion measurements including attempts to escape, attempts to retreat, and gasping were recorded with a video camera. The stunning system containing 90% CO_2_ caused a higher aversion rate when compared to 70% CO_2_, possibly due to severe hyperventilation and increased irritation of the nasal mucosa. Conversely, as the concentration of CO_2_ decreased, time to loss of posture increased, also increasing the perception of the aversive stimulus until consciousness was lost. This study suggested that stunning with CO_2_ is not free from pain or distress since greater concentrations are likely to be needed to achieve timely unconsciousness. Verhoeven et al. [13] evaluated the relationship between onset of unconsciousness and behavioral measurements using 48 crossbred animals exposed in a pre-filled container. Electroencephalogram (EEG) activity was measured and its association with behaviors including sniffing, retreat and escape attempts, muscular contractions and head movements, loss of posture, and gasping were recorded using video cameras. They reported that time to unconsciousness was longer for pigs exposed to 80% CO_2_ when compared with pigs exposed to 95% CO_2_. The 95% CO_2_ group exhibited sniffing, retreat attempts, head movements, and gasping earlier than those exposed to 80% CO_2_. However, all behaviors measured were observed before the loss of consciousness in both treatment groups, and the time to an isoelectric EEG, indicating brain death, was similar for pigs exposed to 80% CO_2_ and 95% CO_2_. 

Kells et al. [21] tested 100% CO_2_, 100% argon, and a 60% argon:40% CO_2_ treatment in pre-weaned piglets. Treatments were administered through a pre-filled plastic 60 L chamber, with five animals per group randomly assigned to a treatment. Prior to the loss of consciousness, piglets exposed to 100% CO_2_ experienced more stress, a longer duration of escape attempts, and labored breathing when compared to piglets exposed to 100% argon; however, among those two treatments, 100% CO_2_ induced the most rapid loss of consciousness and death. Mixing argon with CO_2_ appeared to have no added advantage on the indices of animal welfare examined, including the level of stress and duration of escape attempts.

Llonch et al., 2013 [32], assessed the effect of nitrogen and CO_2_ mixtures in four treatment groups of six commercial female pigs (average live weight of 93 kg) in separate crates. The treatment groups were 70% nitrogen: 30% CO_2_, 80% nitrogen: 20% CO_2_, 85% nitrogen: 15% CO_2_, and 90% CO_2_ in air; all with O_2_ below 2%. Exposure times were varied to provide a short and long duration of exposure within each treatment group. For the nitrogen:CO_2_ mixture groups, the short and long durations were three and five minutes, respectively; the short and long durations for the CO_2_ only groups were two and three minutes, respectively. Results indicated a more rapid loss of consciousness and higher aversive reaction under exposure to 90% CO_2_ in the air compared to nitrogen:CO_2_ mixtures. Furthermore, a significant reduction in brain activity and muscular excitation was observed under the 90% CO_2_ in air treatment when compared to the exposure to all gas combinations. All pigs in the long-duration (three minutes) 90% CO_2_ exposure group died, whereas approximately 75% of animals in the short-duration (two minutes) 90% CO_2_ exposure group recovered consciousness. None of the animals died for nitrogen and CO_2_ mixture groups within the short exposure, and approximately 70% of the animals died after the long exposure, with no differences between the gas mixture compositions. 

The CO_2_: argon combination has also been explored in the literature. Sadler et al. [20] assessed two age groups of pigs, weaned and neonate, in CO_2_: argon gas combination treatments arranged in a factorial design, with two gas types (100% CO_2_ and 50:50 CO_2_:argon) and four flow rates. The flow rates explored were defined as box volume exchange/minute and were classified into slow (20%), medium (35%), fast (50%), and prefill (prefilled followed by 20%). There was also a control treatment in which ambient air was passed through the box. The authors observed animals during both conscious and unconscious phases. Results showed that animal welfare (measured during the conscious phase) and efficacy in causing death were best for the 100% CO_2_ gas treatment and fast flow rates. This was evidenced by lower duration and intensity of stress-related behavior (e.g., open-mouth breathing, righting response, escape attempts) and better efficacy indicators (faster loss of posture and less time to the last movement). The authors also reported that neonates lost consciousness faster, showed fewer signs of distress, and died faster when compared to weaned pigs.

Sutherland et al. [28] evaluated the effect of pig age (one, two, three, four, five, and six weeks of age) and two CO_2_ delivery methods to the containment chamber in two experiments. The first experiment focused on different age groups and included 30 female pigs, five pigs per age group, and utilized a CO_2_ flow rate of 20% chamber volume per minute (gradual exposure). The second experiment focused on CO_2_ delivery and utilized five three-week-old female pigs in a chamber containing 100% CO_2_ with CO_2_ being continuously released to ensure concentrations of at least 90% (pre-filled method). Both experiments collected data regarding latency, panting, open-mouth breathing, righting response, escape attempts, loss of posture, muscular excitation, and respiratory arrest, as well as plasma cortisol concentrations immediately before placement in the chamber and once the heart stopped beating. Results showed no effect of age on behavior responses, loss of posture, or cortisol response when using a gradual flow rate of 20% volume per minute (first experiment). However, there was an increase in time to loss of posture when using gradual filling as compared to the pre-filled container (second experiment). Lastly, an increase in plasma cortisol concentrations and behavioral responses prior to the loss of consciousness were reported across both experiments, regardless of animal age and delivery method. 

Smith et al. (2018) [22] introduced the method of multi-step euthanasia where nitrous oxide (N_2_O treatment) was introduced in a gas chamber for six minutes before CO_2_ was introduced, and compared this method to single-step CO_2_ application in newborn piglets. Piglets in the multi-step N_2_O/CO_2_ treatment took longer to lose posture, and displayed more behavioral signs of stress and aversion including a larger number of escape attempts per pig, squeals/minute, and righting responses. These results indicated that the euthanasia of piglets using the multi-step N_2_O/CO_2_ method was not beneficial nor humane when compared to the use of CO_2_ alone.

Becerril-Herrera et al. (2009) [23] independently compared the effect of CO_2_ and electrocution stunning prior to slaughter by evaluating acid imbalance and blood gas levels. Pigs stunned with CO_2_ showed hypercapnia, hypercalcemia, hyponatremia, hyperglycemia, reduced blood pH (acidemia) with elevated lactate (lactic acidemia), increased hematocrit, and reduced PO_2_, whereas electrically stunned pigs had hyperglycemia, lactic acidemia, and reduced PCO_2_ and PO_2_. The authors suggested that major acid and mineral imbalances seen in CO_2_ stunned pigs as compared to electrically stunned pigs could signal compromising animal welfare prior to death. However, they went on to note that the presence of hyperglycemia and lactic acidemia in both treatment groups indicated pre-sacrifice stress regardless of stunning method.

Meyer et al. (2013) [8] described the effects of a variety of gaseous euthanasia methods including 70:30 N_2_: CO_2_, and 100% CO_2_ under 10% and 20% chamber volume displacement rate, as well as physical methods including electrocution and captive bolt, on stress-related physiological indicators, including plasma levels of cortisol, norepinephrine, and lactate. Results showed similar plasma concentrations for these stress indicators for all treatment groups. Within the inhaled methods (for which unconsciousness is not immediate), time to loss of consciousness and loss of heartbeat was shorter when 100% CO_2_ was used, compared to 70:30 N_2_: CO_2._

#### 3.2.3. Physical Methods

Currently, the American Veterinary Medical Association (AVMA) and the American Association of Swine Veterinarians (AASV) recommend manual blunt trauma as an option for euthanasia of young piglets under three weeks of age [6]. Physical methods that involve impact to the skull with a solid object or surface are the most practical techniques for euthanasia of piglets on farms. However, this method can lack repeatability and accuracy as the success is dependent on the force the stockperson exerts. These limitations may be present if workers are not comfortable with the technique, have not been trained sufficiently to consistently perform the procedure, or if they are not strong enough to adequately carry out the procedure. The method may also be aesthetically unpleasing, which may result in the act of euthanasia being delayed [35].

Casey-Trot et al. (2013) [34] examined the effectiveness of a non-penetrating captive bolt (NPCB) (Zephyr-E, pneumatic device, BOCK Industries, Inc., Phillipsburg, PA, USA) for euthanasia of 100 neonatal piglets younger than 72 h old, performed by ten stockpeople. All piglets (100%) were immediately rendered insensible following application without showing signs of a return to sensibility. Death was achieved without a secondary euthanasia step in 94 of 100 piglets based on a 15 min standard time of observation; four of the six piglets required exsanguination as a secondary step due to the presence of an irregular heartbeat, and two of the piglets required anesthetic overdose as an alternative euthanasia method because of sustained convulsions. Time from application to cardiac arrest differed significantly among stock people, but this did not play a role in effectiveness given that deaths occurred within the 15-min allocated time period. Grist et al. [36] also investigated the use of a mechanical NPCB (Accles and Shelvoke CASH small animal tool, Accles & Shelvoke Ltd., Birmingham, UK) to produce immediate death in neonate piglets when applied in a frontal and parietal position. All 148 piglets in this study demonstrated immediate loss of consciousness, being effectively euthanized immediately. 

Widowski et al. [35] compared the effectiveness of a NPCB device to the traditional method of manual blunt trauma for on-farm euthanasia of low-viability neonatal pigs. In this study, nine stock people performed manual blunt trauma in 76 neonatal piglets and used a NPCB (Zephyr ZE, BOCK Industries, Inc., Phillipsburg, PA, USA) in 99 piglets, with all piglets being randomly assigned to one of the two treatments. For blunt force trauma, all piglets were rendered insensible and heartbeat stopped in less than 3 min, with none showing signs of a return to sensibility. For the NPCB, 13 out of the 99 piglets (13.1%) showed signs of a return to sensibility. Application of the NPCB resulted in a longer duration of leg movement (approximately 124 vs. 68 s) and heartbeat (approximately 409 vs. 171 s) when compared to the manual blunt trauma method. Of note, subcutaneous and subdural hemorrhage scores were greater in NPCB, indicating greater trauma in piglets euthanized by NPCB, but this did not translate to more rapid death or time to final leg movement. Widowski and colleagues [35] concluded that while NPCB effectiveness varied across operators, manual blunt trauma was a rapid, effective, and humane method of euthanizing low viability piglets. 

Lastly, the use of a 12-gauge shotgun for the emergency slaughter or euthanasia of large animals was reported by Blackmore and colleagues [37]. Preliminary trials included isolated heads of two large mature sows (unknown weight) and one large boar (420 kg live weight), followed by one trial using a large white sow (weight reported as 160 kg without the head) placed in a rectangular pen. One of the two sows’ heads was shot using a single 28 g rifled slug (Winchester Super × 1 oz), and the other was shot using buckshot (nine individual lead pellets; Winchester OO/SG), and both were shot in the frontal region. The boar head was shot four times in different positions, and three times using the 28 g slug and one time using the buckshot. The live sow was shot with the buckshot behind the ear. According to the authors, both types of projectiles caused enough damage to the heads to cause immediate insensibility and death in a live animal. Finally, the live sow collapsed into lateral recumbency immediately after the shot, showing pupillary dilation. The animal was ensanguined 30 s after shooting.

Electrocution is another physical method listed as “preferred” by the AVMA for pigs over 4.5 kg. Even though the main drawback for this method is considered to be the need for individualized electrocution, this method has been successfully used for depopulation of approximately 700,000 animals in The Netherlands during Classical swine fever eradication during 1997–1998, when animals were killed outside the barns on an automated device mounted on a truck [39,40].

### 3.3. Small-Scale Studies Using Methods Permitted in Constrained Circumstances 

#### 3.3.1. Ingested/Oral Formulations

Ingestion of sodium nitrate at toxic doses is permitted by the AVMA under constrained circumstances only. Nitrite toxicity induces methemoglobinemia resulting in a rapid decrease of oxygen delivery to the brain as well as vital organs. Pigs are highly susceptible to this toxic mechanism of action, with nitrite causing death within one to one and a half hours following ingestion. Antemortem clinical signs include vomiting, dyspnea, incoordination, paddling, and convulsive seizures lasting less than 30 min [41]. Despite the scarcity of oral formulations for domestic swine, studies show the effectiveness of sodium nitrite to control feral swine populations [42] where other options were deemed not feasible. 

Snow et al. [42] tested a bait containing microencapsulated sodium nitrite incorporated in feed on 56 captive invasive wild pigs. An average of 475 g of toxic bait was consumed per animal resulting in 95% mortality (53 of 56) within three hours, and similar studies have achieved 60–90% mortality rates in feral swine [43,44,45].

#### 3.3.2. Other: Ventilation Shutdown 

The AVMA classifies ventilation shutdown (VSD) as a method for use in the depopulation of pigs under constrained circumstances (Table 1) [1]. Ventilation shutdown includes closing facility openings, shutting inlets, and turning off ventilation fans. Death due to hyperthermia occurs following increases in environmental temperature and humidity resulting from respiration and body heat of the animals. While no experimental or field studies have been published to validate this method, simulations and case studies have found that relative humidity and air temperature increase rapidly within a short period of VSD [46]. Ideally, VSD should only be used in facilities with the capacity to adequately maintain air temperature and relative humidity to a level that results in at least a 95% death rate in less than one hour [1]. While there is no specific information regarding the length of time necessary for VSD to be harmful from the animal welfare perspective, the consensus among experts is that the temperatures that kill will cause suffering. Modified VSD (VSD+) uses an additional heat source(s) or the addition of CO_2_ to achieve the goal of 100% mortality. After VSD/VSD+ depopulation is completed and regular ventilation resumes in the facility, death must be confirmed and a backup secondary euthanasia method applied to any remaining live animals [47].

Historically, ventilation-based depopulation methods have been employed in response to the highly pathogenic avian influenza outbreaks. The USDA approved VSD as the last resort to stamp out infected poultry flocks when standard methods cannot be applied in a timely manner [48]. Nevertheless, the OIE-World Organization for Animal Health guidelines for euthanasia for disease control do not recommend the use of either VSD or VSD+ as they fail to ensure immediate unconsciousness and insensibility or immediate death without distress [49]. Instead, the OIE indicates that VSD and VSD+ cause distress and suffering via prolonged heat stress or conditions for suffocation, and implementation complications can often result in further delays of death. 

### 3.4. Depopulation Methods Scalable to Large Populations

Six large-scale studies assessing different concentrations of CO_2_, argon, and equipment design were identified in the literature [3,24,25,26,27,50]. While Meyer and Morrow [3], as well as Rice et al. [26], focused on equipment design for CO_2_ euthanasia in adult domestic swine, Kinsey et al. [24] focused on the development of a trailer equipment design for feral swine euthanasia. 

Meyer and Morrow [3] conducted two proof-of-concept studies using casualty pigs to examine the use of CO_2_ for swine depopulation: the first used CO_2_ cylinders to dispense liquid CO_2_ with conversion to gaseous CO_2_ within the transfer hose, and the other used low-pressure liquid CO_2_. The first study was conducted in a 14.5 m^3^ dump trailer using a CO_2_ flow rate of 2900 L (20% of the dumpster’s volume) per minute, and 30 pigs. Researchers estimated that the onset of unconsciousness occurred 30 s to one minute following CO_2_ introduction. Most pigs were motionless with slight limb movement at 4.5 min post-administration, and death was confirmed in all (30/30) pigs at 14.5 min. The second study was performed in a smaller, 9 m^3^ dump body trailer for which carbon dioxide was supplied through a liquid-CO_2_ filling box at a flow rate of 19.8 m^3^ per minute at a time rate of 1τ (27.5 s). Two batches of pigs were evaluated, one with 14 and the other with eight casualty pigs. For the first batch, CO_2_ gas was supplied for 90 s (3τ), gas turned off and pigs were allowed to dwell for 15 min. All pigs regained consciousness; therefore, following additional container sealing, CO_2_ was applied for an additional 90 s. After the second administration, 12 of the 14 exposed pigs died after 15-min exposure. Two pigs remained alive but unconscious after the second 15-min CO_2_ exposure and were humanely euthanized. For the second batch, CO_2_ was supplied for 70 s (~2τ), and seven pigs died and one was alive but unconscious after the 15-min exposure. All unconscious live animals were humanely euthanized using a secondary method (penetrating captive bolt). These studies highlighted the importance of assuring that cold liquid CO_2_ is passed through a heat exchanger to avoid freeze-burning and the importance of checking for leaks in the container, which was implicated in the failure in the first attempt in study one and may have contributed to the survival of one pig in batch 2. Both studies justified the importance of confirming death upon finishing the process.

Kinsey and colleagues [24] administered CO_2_ in a portable, self-contained apparatus to feral swine in a three-replicate study. The CO_2_ was administered beginning immediately post-loading, for five minutes at an average of 18% chamber volume per minute. Pigs were monitored for a total of 25 min of CO_2_ exposure. Results indicated 100% mortality rates with an average maximum CO_2_ level of 63% reached in approximately 5.5 min. All animal movement ceased by 10–12 min across replicate. The absence of corneal reflex, rhythmic breathing, response to painful stimuli, movement, vocalization, and death was confirmed in all pigs within the maximum 25-min observation time frame. Both studies indicated that CO_2_ gas, applied at 18–25% chamber volume/minute, was effective when containers were properly sealed. These findings are similar to the AVMA-recommended displacement rate of 20% of chamber volume/minute for 5 min, which has shown to cause 100% mortality and be an effective and predictable method for on-site mass depopulation of pigs [6].

Meyer et al. [27] investigated the use of modified solid waste dumpsters (20- or 30-cubic yard volume) for containment of feeder pigs (mean weights 21.9 to 46.0 kg per trial) during CO_2_ euthanasia in eight trials (6 to 48 pigs per trial, *n* = 212 pigs total). Three of the trials used sublimation of dry ice with externally applied heat and five used low-pressure liquid CO_2_ and a polyethylene storage bladder for storage of gaseous CO_2_ as the CO_2_ source. Time to loss of consciousness was observed via video and measured as a loss of righting reflex. The median time to unconsciousness was 84 and 120 s from the start of CO_2_ delivery for the first pig and for all pigs, respectively. This study showed that on-farm depopulation could potentially be conducted using common equipment, supplies, and little training. However, it is important to note that the rapid generation and storage of CO_2_ would be required for the rapid depopulation of a single, large group of pigs, which could be a challenge for large facilities.

Stikeleather et al. [25] and Rice et al. [26] focused on describing CO_2_ distribution requirements for on-farm euthanasia of pigs. Stikeleather et al. [22] used computational fluid dynamics to better understand CO_2_ gas dynamics within the chamber. Results showed that CO_2_ concentrations on a horizontal plane at the approximate height of pig’s nostrils (25 cm from the floor) were uniform over a 5-min period, which indicated that a plenum was not needed for these types of euthanasia procedures. Furthermore, the analyses highlighted that the 20% volume per minute of CO_2_ inflow rate could potentially be reduced when considering the volume of the pigs in the chamber and the uniformity of gas achieved within the chamber, which decreased the need for an excess chamber height. Lastly, results showed that chamber leaks on the top part of the chamber were less critical as compared to leaks in the bottom and/or between walls. Rice et al. [26] provided a detailed description of a methodology for CO_2_-related euthanasia for small groups of pigs using a low-cost, high-pressure CO_2_ cylinder and a small dump-style trailer. A detailed diagram of CO_2_ application to the chamber, along with a prototype for the application system and a list of materials needed, is provided. The authors concluded that CO_2_ delivery systems can be successfully set up on-farm and that the only limiting factor for timely large-scale euthanasia would likely be the size of the chamber and the ability to provide an appropriate CO_2_ flow.

Apart from AVMA preferred methods, Fiedler et al. [50] evaluated alternative gases, specifically argon gas, for euthanasia of weaned cull pigs (3 to 16.6 kg). This study did not support argon gas euthanasia as a method of improving animal welfare. In this study, 223 weaned pigs were randomly assigned to group sizes of one, two, or six pigs placed into the argon pre-filled chamber with oxygen less than 2% volume. The stocking rate was shown not to affect the onset of neuromuscular excitation or last movement. Solitary pigs in a chamber were more likely to pace and make righting attempts when compared with multiple pigs in the chamber. Conversely, pigs in higher stocking rate treatments tended to retain posture longer. These findings disagree with Raj et al. [18,19], which had reported benefits from the welfare standpoint upon the addition of argon. Some reasons that might explain the different results observed between these studies include differences in animal age and health status.

### 3.5. Important Considerations for Depopulation Studies 

A limited number of studies focused on measures of unconsciousness, insensibility and death, ease of carcass disposal, human safety risk, animal welfare, personnel skill, equipment, and facilities requirement. Sadler et al. [20] used corneal reflex response, pupillary reflex, and nose prick as insensibility tests and auscultation to confirm the absence of heartbeat as death confirmation in weaning pigs. In this study, 75% of the pigs (*n* = 180) did not achieve the last movement during the initial ten minutes of gas application of the slow flow rate 50 CO_2_:50 argon gas treatment. Of these, 47% of pigs were still sensible, and blunt-force trauma was used immediately for humane euthanasia. Methods including blunt-force trauma and non-penetrating captive bolt are aesthetically unpleasant for both the operator and any bystanders [51,52].

A summary of human safety risks, skills required, and aesthetics for AVMA preferred and preferred under constrained conditions methods and their respective limitations of the methods as stated by the National Pork Board and OIE are summarized in Table 2 [47,53]. Recommendations from the National Pork Board suggest that gunshot has the highest human safety risk, while injectable anesthetic overdose, non-penetrating captive bolt, and manual blunt force trauma have low human safety risks. The skill required across these methods was also rated, with the use of injectable anesthetics requiring the highest amount of expertise. 

## 4. Discussion

According to the United States Department of Agriculture (USDA)’s National Agricultural Statistics Service, the US swine inventory was 79.6 million head as of 1 June 2020 [54], with the vast majority of US swine reared indoors in modern facilities under controlled environments. Even though indoor housing, outdoor housing, and free-ranging feral swine all pose unique challenges when depopulation is needed, large farms using indoor housing pose a special challenge owing to a large number of swine on the site, as well as the presence of slatted floors and deep manure pits; these features make some euthanasia methods (e.g., CO_2_ inhalation) non-feasible and present significant challenges to post-death handling and disposal [6]. These direct and indirect factors should be well noted when considering future studies for swine mass depopulation. Injectable, physical, and ingestion/oral methods studied have been applied at the individual or small group levels. They do not appear to be good candidates for mass depopulation in swine because they would be time-consuming and/or unsafe for animals as well as operator personnel. They may also be minimally suitable considering animal welfare and potentially negative emotional impacts on personnel. Physical methods involving the discharge of blood or body fluids also may pose an unacceptable risk of disease dissemination among other domestic swine populations as a result of pathogen dissemination during the movement of carcass material to disposal sites [55].

The major findings of this systematic review are that there are few studies on the topic of mass depopulation of swine, available reports are inconsistent in describing main characteristics of depopulation methods, and most assume a scalable process based on small sample sizes. Future studies would ideally include, as a minimum: the number and age of pigs included in the studies, measures of unconsciousness, time to loss of consciousness, death and insensibility confirmation methods, ease of carcass disposal, human safety risk assessment, animal welfare assessment and issues, personnel skill and labor requirements, and equipment and facilities requirements. Furthermore, in the present literature, there was a degree of subjectivity and lack of standardization for important characteristics such as determination of the time to death. This is not an uncommon issue even considering the human medicine field [56], but for the purposes of depopulation, once unconsciousness occur, adverse welfare is no longer an issue as long as the process is irreversible and shown to reliably result in death. 

The lack of standardization for the above-mentioned metrics, combined with the variety of possible method combinations examined in a single study from the ones examined reduced the number of direct comparisons among studies that could have been done. As an example, from the 18 inhaled methods-based studies discussed in this review, only half of those provided a clear metric for how unconsciousness was measured, and approximately 60% of them provided a clear description of welfare-based assessments. Even when examining the 60% that did provide clear assessments, these were highly variable, with authors including anywhere from a measure of “public perception” by experts [33] and animal “choice” to stay in certain environments [18], to quantifications of stress indicators in the plasma [8], escape attempts and distress [19], lateral recumbence [27], among others. This limited comparisons across studies. 

Inhalation-based depopulation using gaseous CO_2_ was the most commonly investigated method in the literature. This was not surprising given there is a substantial body of literature supporting the uses of CO_2_ for slaughter stunning in swine [11,57,58,59,60]. However, it is very important to note that meat-packing CO_2_ systems are designed based on research findings under controllable conditions within a plant, whereas on-farm depopulation scenarios are considered highly variable due to extraneous factors. In addition, distress, aversion, and escape behaviors in response to the presence of a high concentration (greater than 50%) of CO_2_ in the air indicates that CO_2_ can compromise pig welfare due to air hunger, fear, anxiety, and pain [16]. There is evidence of CO_2_ causing direct painful sensation through the trigeminal nerve in humans as well as other experimental animal models such as rats [61,62]. However, researchers have not consistently observed this effect [18], and some found CO_2_ concentrations as high as 90% to be considerably less aversive than electrocution [63]. These differences may be attributable to different concentrations of gases, age of animals, time of gas exposure, variability in the genetics of the animals studied, and types of behaviors assessed [28,64]. For instance, in swine, Duroc pigs showed more aversion to 90% CO_2_ in comparison with Large White pigs in one trial; however, another trial by the same authors reported the difference to be attributable to individual animal temperament rather than a breed effect [17,18]. Combinations of CO_2_ with inert gases such as argon were most commonly investigated to reduce aversion to CO_2_, and these studies appeared to be promising in reducing the averseness caused by CO_2_ alone [17,65].

A few scaled studies, which mainly focused on equipment design [3,24,25,26,27], addressed other practical concerns including sources of gases and costs. Meyer et al. [27] indicated the possibility of CO_2_ production by heat sublimation of dry ice or by conversion of low-pressure liquid CO_2_ to gas prior to each test using exogenous environmental heat. Meyer et al. [27] also stated that options for equipment may consist of common items that may be found on site or that are readily available from farm supply stores, often requiring no special training to assemble or implement. These are important considerations when focusing on mass depopulation preparedness.

Besides a few CO_2_ trials, effectiveness studies under field conditions and in groups with large numbers of swine were largely lacking in the literature. Future studies are needed to support large-scale emergencies requiring multiple animals to be euthanized in a short window of time (24 h or less). A few emerging technologies that have been described for poultry or cattle but not extensively for swine include low-atmospheric-pressure stunning (LAPS) [6] and Diathermic Syncope^®^ (DTS). The only report describing a decompression system for swine has been described in the grey literature [33], in which a cylinder chamber was used in small groups of pigs (1 and 3) that were 30kg and smaller. In this case, a vacuum pump and pressure control systems were used. Results were promising, with the period from the first sign of reaction to the last movement of animals being from 9 to 15 min [33]. The DTS system has been recently described for slaughter stunning of cattle [66], but there are no reports of its use in swine. Furthermore, the use of compressed air foam systems (CAFS), has been reported to be effective in the depopulation of large numbers of caged layer hens [67], but there are no reports for their uses in swine. Methods such as water-based foam are not approved for swine by the AVMA, and animal welfare critics claim CAFS fails to ensure immediate unconsciousness and insensibility or immediate death without distress [68]. In addition, for pig welfare and practical personnel implications on the farm, it is critical to reduce the number of pigs that require a secondary euthanasia step. Of important note, once an animal is unconscious, the point of interest shifts from welfare to efficacy since removing animals from sites without risk of disease spread or extended personnel efforts are important in depopulation scenarios. As such, it is vital that the process is practical for on-farm implementation as stated on the AVMA guidelines for euthanasia and depopulation [6,24,25,26,27,69]. In addition to identifying a preferred method for swine depopulation, it is important to consider the knowledge, empathy, and experience of individual caretakers and their attitudes toward decisions [23,70,71]. Information on this topic was largely lacking considering all the studies assessed in this review.

Apart from domestic pigs, the demand for population reduction for feral pigs has also increased over the years. Feral swine in the US has spread from nine states in the 1980s to more than 44 states in 2010, causing an estimated economic impact of nearly $1.5 billion annually in crop damages and control costs in the U.S. [72]. Feral pigs also spread diseases, damage ecosystems by reducing plant species diversity, depredate sensitive species, and destroy habitats of desired native species [73]. Although various toxicants including sodium fluoroacetate, yellow phosphorus, and warfarin have been used to control feral pigs, sodium nitrite appears to be more acceptable in terms of persistence in the environment and humaneness concerns. However, effectiveness has been shown to be majorly dependent on bait acceptance [44]. The potential for secondary transfer to non-target species is reduced since most mammals including humans, domestic pets, and livestock (except domestic swine) produce sufficient amounts of the enzyme necessary for reduced sensitivity to low doses of sodium nitrite. Sodium nitrite is still under evaluation by the Environmental Protection Agency to review the potential risks to human health [42]. 

Euthanasia of individual animals or mass depopulation may be inevitable and necessary. The recent rise in public awareness about livestock mass depopulation conditions has become a subject for great discussion among animal rights groups, veterinary communities, and the overall public. Of note, during the year 2020, the World Animal Protection [74] published a letter urging AVMA to immediately remove ventilation shutdown from their guidelines so they will not be used to cull or ’euthanize’ farm animals using this method. This specific letter highlights that there is no data supporting these methods to cause immediate unconsciousness and insensibility or immediate death without distress. Transparency and public education in regards to this issue will likely need to be considered in cases where swine depopulation is necessary, and appropriately communicating with different audiences on the subject will be an important piece of the process.

## 5. Conclusions

In conclusion, we reviewed research from the past 30 years on methods for depopulation of swine. The use of CO_2_ is the commonly reported method in literature, with higher concentrations causing faster unconsciousness at the expense of compromised welfare expressed by the provocation of air hunger, anxiety, and fear. Despite research over three decades, a safe and reliable way to induce rapid unconsciousness and death in larger populations of swine appear to have not been found.

During our attempt to systematically review methods and evaluate studies, we identified inconsistency in metrics on unconscious states and animal welfare features. This major challenge prohibited us from conducting quantitative comparisons of depopulation methods that could aid in providing specific recommendations to producers, educators, and other livestock industry stakeholders. Therefore, there is an urgent need to standardize comprehensive reporting guidelines for depopulation studies. Such reporting should include the number of animals per experiment, quantitative measurements including speed of loss of consciousness, death and insensibility confirmation, and human safety risk assessment. Standardization of such reporting would increase the utility of future studies seeking to improve animal welfare and personal safety during depopulation procedures.

## Figures and Tables

**Table 1:** List of preferred swine depopulation methods as well as those permitted in constrained circumstances.

Age Category	Method	Preferred	Permitted in Constrained Circumstances
All ages	Injectable	Anesthetic overdose, intravenous barbiturates and barbituric acid derivatives	Expired, compounded, non-pharmaceutical grade injectable euthanasia agents or anesthetics
	Inhaled/gas	Carbon dioxide	
	Physical methods	Gunshot to the head (not appropriate for depopulation of suckling pigs), penetrating captive bolt, electrocution	
	Ingested/Oral		Sodium nitrate
	Other		Ventilation shutdown (VSD) or VSD plus
Only for suckling and young pigs	Physical methods	Manual blunt force trauma, non-penetrating captive bolt	

Source: AVMA Guidelines for the Depopulation of Animals [1].

**Table 2:** Summary of human safety, required personnel skill, aesthetics, and limitations of different depopulation methods for swine (adopted from the National Pork Board’s guidelines “On-Farm Euthanasia of Swine Recommendations for the Producer” and from OIE guidelines for ‘’Killing of animals for disease control purposes’’).

	National Pork Board (NPB), 2016 [47]					OIE, 2019 [53]	
	Human Safety Risk	Skill Required	Aesthetics	Limitations	Approved for	Restraint Necessary	Animal Welfare Concerns If Applied Inappropriately
Carbon dioxide (CO_2_)	moderate	moderate to low, based on equipment design	bloodless, some excitatory movement or vocalization	practical for small pigs only; maintenance of equipment	all ages (may not be practical for pigs over 70 pounds (31.7 kg))	yes	slow induction of unconsciousness, averseness of induction
Gunshot	high	moderate to high	discharge of blood from wound	security of firearms; legal restrictions; maintenance of equipment	nursery pigs or older	no	non-lethal wounding
Non-penetrating captive bolt	low	low	minimal to some blood discharge as a 1-step process	may be a 2-step process based on equipment design and size of pig; maintenance of equipment	pigs less than 70 pounds (31.7 kg)	yes	non-lethal wounding
Penetrating captive bolt	moderate	moderate	discharge of blood from wound	may be a 2-step process depending on equipment design; maintenance of equipment	pigs greater than 12 pounds (5.4 kg)	yes	ineffective stunning, non-lethal wounding, regaining of consciousness before death
Electrocution (head-only and head-to-heart)	low if proper lock out/tag out procedure followed	moderate	muscle contraction; minimal to no blood discharge	adequate amperage and voltage needed; head only is a 2-step process; cleanliness of electrodes	pigs over 3 days of age	yes	pain associated with cardiac arrest after ineffective stunning; design of the stunning tongs not appropriate for the small head or body of neonates
Veterinarian administered anesthetic overdose	low	high, veterinary administration only	no blood discharge, limited pig movements	applicable agents available only to licensed veterinarian; carcass disposal	all ages but may not be practical	yes	non-lethal dose, pain associated with injection site
Manual blunt force trauma	low	moderate	some blood discharge; objectionable for some	only applicable to small pigs; ability of caretaker to apply sufficient force	pigs up to 12 pounds (5.4 kg)	yes	non-lethal wounding
